# Expanded Education, Better Health in Later Life? –Evidence from the Mexican Health and Aging Study (MHAS)

**DOI:** 10.1093/geroni/igaf122.2668

**Published:** 2025-12-31

**Authors:** Chengming Han, Brian Downer, Zhiwei Hu

**Affiliations:** UTMB Health, The University of Texas Medical Branch, Galveston, Texas, United States; UTMB, Galveston, Texas, United States; the University of Texas Medical Branch, Galveston, Texas, United States

## Abstract

**Objective:**

ObjectiveThis study intends to examine the education gradients in functional limitations for older adults in Mexico by comparing effect size between two birth cohorts age 60-76 in 2001 versus 2018.

**Methods:**

Data were drawn from the Mexican Health and Aging Study (MHAS) 2001 and 2018 waves. Tobit model was used to estimate the education gradient in functional limitations. Living conditions in childhood (pluming in house, parents’ education, and serious health conditions), chronic conditions, and the longest work were adjusted for. The cohort difference was tested by including interaction effects and propensity matching scores.

**Results:**

Compared to the 2001 cohort, the 2018 cohort presented lower proportion of no education, and higher proportion in each level of education, especially the secondary and tertiary education. Education gradients were significant in functional limitations for each birth cohort, but the education gradient was attenuated for the 2018 cohort compared to the 2001 cohort. Living conditions in childhood have long-term effects on functional limitations in later life. Agricultural and industrial work contribute to greater functional limitations. No different effects of primary job/work and early life conditions between the cohorts were detected.

**Conclusion:**

Disregarding the average higher levels of education within the 2018 wave birth cohort, the education gradients in functional limitations decreased compared to the early birth cohort in 2001 wave.

